# Determinants of HIV-1 drug resistance in treatment-naïve patients and its clinical implications in an antiretroviral treatment program in Cameroon

**DOI:** 10.7448/IAS.17.4.19615

**Published:** 2014-11-02

**Authors:** Alexander Zoufaly, Johannes Jochum, Raffaela Hammerl, Nilofar Nassimi, Yurika Raymond, Gerd-Dieter Burchard, Stefan Schmiedel, Jan-Felix Drexler, Campbell Naomi Kay, Nchang Taka, Charles Awasom, Karin Metzner, van Lunzen Jan, Torsten Feldt

**Affiliations:** 1 Medical Department, Kaiser Franz Josef Hospital, Vienna, Austria; 2 Medical Department, University Medical Center Hamburg-Eppendorf, Hamburg, Germany; 3 Clinical Research Unit, Bernhard Nocht Institute for Tropical Medicine, Hamburg, Germany; 4 Day Hospital at Regional Hospital Bamenda, Bamenda, Cameroon; 5 Institute for Virology, University of Bonn, Bonn, Germany; 6 Division of Infectious Diseases, University Hospital Zurich, Zurich, Switzerland; 7 Hospital Epide, Zurich, Switzerland; 8 Medical Department, Regional Hospital Bamenda, Bamenda, Cameroon; 9 Infectious Diseases Unit, University Medical Center Hamburg-Eppendorf, Hamburg, Germany; 10 Clinic for Gastroenterology, University Hospital Duesseldorf, Duesseldorf, Germany

## Abstract

**Introduction:**

Facing the rapid scale-up of antiretroviral treatment (ART) programs in resource-limited settings, monitoring of treatment outcome is essential in order to timely detect and tackle drawbacks [1].

**Methods:**

In a prospective cohort study, 300 consecutive patients starting first-line ART were enrolled between 2009 and2010 in a large HIV treatment centre in rural Cameroon. Patients were followed up for 12 months. Virologic failure was defined as a VL >1000 cop/mL at month 12. Besides CD4 and viral load (VL) analysis, HIV-1 drug resistance testing was performed in patients with VL>1000 copies (c)/mL plasma. In those patients and controls, minority HIV-1 drug resistance mutations at baseline, and plasma drug levels were analyzed in order to identify the risk factors for virologic failure.

**Results:**

Most enrolled patients (71%) were female. At baseline median CD4 cell count was 162/µL (IQR 59-259), median log10 VL was 5.4 (IQR 5.0–5.8) c/mL, and one-third of patients had World Health Organisation (WHO) stage 3 or 4; 30 patients died during follow-up. Among all patients who completed follow-up 38/238 had virologic failure. These patients were younger, had lower CD4 cell counts and more often had WHO stage 3 or 4 at baseline compared to patients with VL<1000c/mL. Sixty-three percent of failing patients (24/38) had at least one mutation associated with high-level drug resistance. The M184V mutation was the most frequently detected nucleoside reverse transcriptase inhibitor (NRTI) mutation (n=18) followed by TAMs (n=5) and multi-NRTI resistance mutations (n=4). The most commonly observed non-nucleoside reverse-transcriptase inhibitor (NNRTI) resistance mutations were K103N (n=10), Y181C (n=7), and G190A (n=6). Drug resistance mutations at baseline were detected in 12/65 (18%) patients, in 6 patients with and 6 patients without virological failure (p=0.77). Subtherapeutic NNRTI levels (OR 6.67, 95% CI 1.98–22.43, p<0.002) and poorer adherence (OR 1.54, 95% CI 1.00–2.39, p=0.05) were each associated with higher risk of virologic failure in the matched pair analysis. Unavailability of ART at the treatment centre was the single most common cause (37%) for incomplete adherence in these patients.

**Conclusions:**

Virologic failure after one year of first-line ART in rural Cameroon was not associated with transmitted drug resistance, but with reduced drug plasma levels and incomplete adherence. Strategies to assure adherence and uninterrupted drug supply are important factors for therapy success.
